# Digital health in perinatal care: Exploring usage, attitudes, and needs among Swiss women in urban and rural settings

**DOI:** 10.1177/20552076241277671

**Published:** 2024-09-02

**Authors:** Stephan Oelhafen

**Affiliations:** School of Health Professions, 69477 Bern University of Applied Sciences, Bern, Switzerland

**Keywords:** Digital health, eHealth, Switzerland, attitudes, perinatal care, pregnancy, postpartum‌, rurality

## Abstract

**Background:**

Switzerland's healthcare system is known for its quality but faces challenges such as slow digitalization and fragmentation, especially in perinatal care. This study investigates Swiss women's use, needs, and attitudes in respect of digital health tools during pregnancy and postpartum, focusing on any differences between rural and urban populations.

**Methods:**

A web-based cross-sectional survey targeted pregnant women and those who had given birth in the last 12 months. Participants were recruited through social media, and the data were analyzed using principal component analysis and multivariable regressions to explore factors affecting the use of digital tools and attitudes toward eHealth.

**Results:**

A total of 1160 participants completed the survey. Healthcare professionals (92%) and private networks (77%) were the primary sources of information. Women expressed a strong preference for app features such as data access (73%), prescription management (73%), and scheduling appointments with healthcare professionals (71%). However, they also raised concerns about the impersonal nature of digital healthcare interactions (71%). Overall, rural women had more negative attitudes toward online health information seeking, which can be attributed to differences in education levels.

**Conclusion:**

The findings indicate that while Swiss women in the perinatal period do utilize digital tools, they focus more on nonmedical topics such as tracking physiological development. The study underscores the importance of adapting digital health solutions to the specific needs of women in the perinatal period. Emphasis should be placed on developing applications that are not only informative but also empower women on their healthcare journey while ensuring data privacy and supporting personal interactions with healthcare providers.

## Introduction

Switzerland is known for its high-quality healthcare system; according to the Organisation for Economic Co-operation and Development, the country performs well on various relevant indicators of health status and quality of care.^1,2^ Yet, despite the overall high quality, the Swiss healthcare system also faces significant challenges, including low economic efficiency and a high level of fragmentation in terms of both healthcare provision and documentation.^3–5^ This fragmentation is one consequence of a strongly federalist system, in which the 26 cantons^a^ have substantial autonomy with regard to their healthcare system, federal laws and regulations, private health insurance companies, and associations of healthcare professionals.^6,7^ Although this fragmentation does not seem to significantly affect the overall quality of healthcare, it introduces considerable challenges in system efficiency and coordination. Due to system fragmentation and resistance among healthcare professionals—with some professions and settings exhibiting stronger resistance than others—the digitalization of Switzerland's healthcare system lags behind other nations, and a significant proportion of outpatient healthcare providers still use paper-based patient records.^1,6,7^

In the context of obstetrics in Switzerland, the care model is even more characterized by fragmentation, particularly at the transitions between pregnancy, childbirth, and postpartum care. In Switzerland, prenatal care is delivered mainly by gynecologists in outpatient settings, and most births (≈97%) occur in hospitals. On the other hand, postpartum care is primarily administered through home visits by midwives. These frequent changes in terms of primary healthcare providers and transitions from outpatient to inpatient settings (and vice versa) are inefficient and pose a significant risk of disrupting the flow of information. This is especially the case for women with high-risk pregnancies or polymorbid pathology, who must see an even larger number of healthcare providers. Accordingly, a robust system for data transmission is required to ensure comprehensive healthcare, making the delayed adoption of electronic health records even more relevant.

During the COVID-19 pandemic, there was a significant increase in the use of digital tools, mainly in outpatient settings or at home. In Switzerland, the proportion of physicians using video connections rose from one-10th to about a quarter.^8^ However, one report from Germany showed that while videoconferencing was considered the most suitable form of digital healthcare delivery, a majority of midwives used phone calls or chat services to communicate with their clients.^9^ In general, although many midwives wanted to retain the option of digital care after the pandemic, they emphasized that this should not replace home visits, as it limited their ability to perform examinations or assess the overall situation.^9,10^ Furthermore, midwives in Switzerland are not routinely reimbursed for telephone consultations or telemedical applications, although some exceptions were made during the COVID-19 pandemic.^11^

While the use of digital tools in Switzerland increased during the COVID-19 pandemic, the vast majority of professionals and the general population believe that only a fraction of their full potential has been realized.^8,12^ Although an increasing number of healthcare professionals believe that eHealth has great potential, many midwives and physicians are still reluctant to use digital tools more frequently (cf.^8,13^). Compared to healthcare professionals, the general population seems more open to the use of digital health applications; one representative Swiss survey reported that about 60% of the public would be interested in using remote monitoring of vital signs in cases of chronic illness as compared to about 30% of physicians.^12^ A majority would be willing to allow healthcare professionals to access their data, and three-quarters regard electronic health records as a good thing.^8^ In this context, it is crucial to note that women in the perinatal period are especially predisposed to use eHealth applications more frequently, as 95% use the internet daily and use smartphones to access health data during pregnancy or after delivery.^14–17^ Pregnancy apps that provide information and monitor personal data are also the dominant genre in digital health.^18^

Given the lag in digitalization of the Swiss healthcare system and the ongoing reluctance of some healthcare professionals, the interest shown by women may afford an opportunity to promote eHealth services and tools. It is therefore crucial to better understand the purposes for which women use digital health applications, their attitudes toward digitalization, and what additional functionalities they would like to see. It would also be interesting to know more about the sociodemographic profile of eHealth adopters; for example Plugeisen and Mou^19^ found that women whose pregnancy was not their first preferred virtual care—a combination of in-clinic visits and videoconferencing—rather than traditional care. In addition, attitudes to eHealth and telemedicine may differ depending on whether a woman lives in a rural or an urban area. For instance, telemedical applications that monitor fetal heart rate can avoid the need for longer journeys and facilitate embedded care in private surroundings (cf. Saad et al.,^20^ van den Heuvel et al.,^21^ and Wang^22^). On the other hand, women in rural areas may be more reluctant to use eHealth technologies.

To address these questions, the present study sought to investigate women's needs when using digital tools and what functions these tools currently fulfill in telemedical obstetric care. A further aim was to explore women's attitudes to eHealth applications, including any reservations or limitations. Finally, the study explored the attitudes of rural women to telemedicine and how these might differ from those in urban areas. The study's overall aim was to shed light on the potential role of eHealth in Switzerland for women in the perinatal period and for the rural population.

## Methods

### Study design

A web-based cross-sectional survey study was conducted using a self-administered questionnaire. This forms part of the Smarter Health for Peripheral Regions (SHAPIRO) project, which aims to assess the needs and attitudes of users and healthcare professionals in respect of eHealth applications in the perinatal period. The iterative development of a platform or app based on these data seeks to optimize the efficiency, quality, and/or costs of obstetric care.

### Study population and recruitment

The target population included women aged at least 18 years who were pregnant or had given birth in the last 12 months; there were no other inclusion criteria. As the aim was not to report precise population estimates but to explore the needs and use behaviors of pregnant and postpartum women in respect of digital tools, we relied solely on social media platforms for recruitment. For that reason, we did not seek to ensure that the sample was representative of the target population, and there was no attempt to determine a required sample size a priori. However, we compared our sample with census data across multiple variables to assess the extent of any nonrepresentativeness.

The recruitment ads on Facebook and Instagram included a short video close-up of a pregnant woman rubbing her hands over her belly (AdobeStock video #248958510) and another video of a mother's hand rubbing a newborn's head (AdobeStock video #238836093). To avoid any bias favoring women with an interest in digital tools or apps, the ads used neutral language, asking only if the woman was pregnant or had given birth in the past year. Additionally, the consent page stated that we wanted to better understand how women inform themselves about topics related to pregnancy, birth, and the postpartum period.

The Facebook and Instagram recruitment ads were shown to German-speaking women in Switzerland aged 18 to 45 years, and recruitment took place in one wave over three weeks in June 2022. The advertising budget for the survey was initially set at 150 Swiss Francs; once the first wave ended, the sample of more than 1100 completed responses was considered sufficiently large for present purposes.

### Questionnaire

The questionnaire was designed to explore why and how frequently women use digital sources of information related to pregnancy, childbirth, and the postpartum period. Additionally, we aimed to explore general attitudes to digital tools and any desired features. Based on a literature review, an initial draft of the questionnaire was developed. The initial set of items was derived from or inspired by existing questionnaires exploring demographic and pregnancy- and childbirth-related variables, including online health information-seeking behavior and topics of interest to women during the perinatal period.^23–29^ Some items from existing scales were translated and adapted for the purposes of the current study. The initial draft, which included 35 items, was validated using the Content Validity Index.^30^ Six colleagues, including external experts in midwifery, gynecology/obstetrics, and healthcare digitalization, were briefed on the study context and objectives and were asked to rate the draft questionnaire items in terms of clarity and relevance. The average item clarity rating was M = 0.78 (range 0.33–1.00), and the average relevance rating was M = 0.97 (range 0.80–1.00). Given the small sample of experts, we used qualitative feedback to improve the clarity of 11 low-rated items.

The final questionnaire (see Supplemental Table S1) included six items assessing pregnancy or birth-related variables, women's use of digital media in general (one item), online health information-seeking behavior (one item), use of digital tools, websites, apps, and forums related to pregnancy or motherhood (eight items), attitudes to various hypothetical functionalities and healthcare system digitalization in general (four items), and sociodemographic information (eight items). The questionnaire was implemented using LimeSurvey (Version 2.56.1), focusing on display quality on mobile phones. All response options were displayed randomly unless they entailed a natural order (e.g. age categories).

### Data analysis

The primary objectives of the analysis were (a) to describe current usage frequencies and attitudes to eHealth applications and (b) to identify the factors underlying use behaviors and attitudes. To address the latter issue, we used the Psych package to perform a principal component analysis (PCA), condensing the range of items into fewer interpretable components.^31^ To assess the suitability of the data for PCA, we utilized Bartlett's test of sphericity and the Kaiser–Meyer–Olkin (KMO) measure of sampling adequacy. Additionally, we used parallel analysis to determine the appropriate number of components to be extracted. In the case of items involving binary responses (e.g. “True/False”), polychoric correlations were computed before the analysis to ensure the validity of the results.

Predictors associated with the outcome variables of interest were estimated using multivariable regression models, taking account of typology (urban/intermediate/rural region), phase (pregnancy/motherhood), parity (primiparous/multiparous), high-risk pregnancy (true/false), and having a university degree (true/false) while also controlling for age category (see Table 1), married (true/false), and insurance (semi-/private/general). Crude estimates from univariable regression and adjusted estimates from multivariable regressions provided a nuanced understanding of the relationship between these factors and outcomes. Variables with arbitrary numerical scales were standardized to improve the comparability of predictors. We set the alpha level for all statistical tests at .05 to assess the significance of our results. All statistical analyses were performed using R 4.3.1.^32^

**Table 1.** Survey participants: Demographic characteristics (*N* = 1160).

Characteristic	*n* (%)
Age	
under 20	0 (0)
20–24	15 (1.3)
25–29	197 (17)
30–34	591 (51)
35–39	282 (24)
40–44	69 (6.0)
45 or older	4 (0.3)
Marital status	
Married/registered partnership	851 (74)
Single	294 (25)
Divorced/widowed/other	12 (1.0)
Education	
Compulsory education	14 (1.2)
Apprenticeship	292 (25)
Grammar school, Vocational A level, Specialized secondary school certificate (FMS), Vocational college (DMS)	55 (4.8)
Higher technical and vocational training	309 (27)
University of Applied Sciences, educational college	293 (25)
University, Federal Institute of Technology (EPFL, ETH)	190 (16)
Other	4 (0.3)
Health insurance	
General coverage	937 (81)
Semiprivate	180 (16)
Private	36 (3.1)
Unknown	6 (0.5)
Typology	
Urban	559 (49)
Intermediate	273 (24)
Rural	310 (27)
Travel time ≥30 min	81 (7.0)

One researcher classified all the responses to open text questions and “Other” options and standardized the different spellings of apps, forums or blogs, websites, and maternity health records to enable an evaluation of frequencies.

To assess the representativeness of our sample, demographic and selected birth-related variables were compared to multiple census datasets from the Swiss Federal Statistical Office.^33^ Participants were categorized on the basis of the postal codes provided and the Swiss Federal Statistical Office's spatial typology,^34^ assigning them to urban, intermediate (periurban), and rural residential areas.

The study follows the Consensus-based Checklist for Reporting of Survey Studies (CROSS).^35^

### Ethical considerations

The study was reviewed by the Bern University of Applied Sciences Review Board (EAB2022_008) and the Ethics Committee of the canton of Bern. The review confirmed that, under the Swiss Human Research Act, the study was exempt from full ethical assessment (Req-2022-00612). On the first page of the questionnaire, participants were assured of the anonymity and confidentiality of the collected data. By clicking on a checkbox, they indicated that they understood the study's objectives and provided their informed consent. The survey data were stored on the University's designated LimeSurvey server and (for subsequent data analysis) on servers requiring two-factor authentication.

## Results

### Survey responses

The social media link to the survey was clicked 2206 times. Of the 1640 women who provided informed consent, 45 had to be excluded because they did not match the inclusion criteria or did not provide the requisite information about being pregnant or giving birth within the previous 12 months. Of the remaining respondents, 1160 (72.7%) completed the questionnaire in full. To assess the randomness of missing responses, we compared participants who completed the questionnaire with those who dropped out across 14 variables (demographic factors, pregnancy, childbirth, and postpartum characteristics). The only significant difference related to primary pregnancy care; of those who completed the survey, 62 women (13%) were cared for primarily by midwives as compared to 10 women (6.8%) in the dropout group (Fisher's exact test, *p* = .016). With the exception of responses to open-ended qualitative questions, the rate of missing data was less than 1% for all variables among those participants who completed the survey in full. Based on the combined demographic data, no duplicates were identified.

### Characteristics of the study participants

Descriptive demographic statistics can be found in Table 1, and relevant pregnancy- or birth-related variables are shown in Table 2. The proportion of women under 30 years was slightly lower in our sample (18%) than in the census data (25%).^33^ However, our sample was comparable to the census in terms of women holding a university degree,^36^ women working part- or full-time,^37^ and married women.^38^ The proportion of women living in rural areas was higher in our sample (27%) than in the census (16%).^39^ When asked about travel time to the nearest hospital, only 7% of our respondents indicated that it would take them 30 min or more.

**Table 2.** Survey participants: Selected pregnancy- and birth-related variables.

Characteristic	Pregnant women (*n *= 490) *n* (%)	Postpartum women (*n *= 670) *n* (%)	*p* ^a^
Primiparous	263 (54)	395 (59)	.065
Gestational age (weeks) (mean (SD))	25.9 (8.9)		
Child's age (months) (mean (SD))		5.9 (3.6)	
Mode of delivery (*preference*)			
Vaginally	436 (89)		
Cesarean section (on medical advice)	31 (6.3)		
Cesarean section (by personal choice)	22 (4.5)		
Mode of delivery (*factual*)			
Noninstrumental vaginal birth		394 (59)	
Forceps or vacuum birth		65 (9.7)	
Planned Cesarean section (on medical advice)		93 (14)	
Planned Cesarean section (by personal choice)		14 (2.1)	
Unplanned Cesarean section		104 (16)	
High-risk pregnancy	111 (23)	187 (28)	.043
Main caregiver (pregnancy)			.2
Obstetrician/gynecologist	418 (85)	566 (85)	
Midwife	62 (13)	97 (15)	
Practitioner	5 (1.0)	3 (0.4)	
Other	5 (1.0)	2 (0.3)	
Place of birth (preferred/actual)			<.001
In a hospital	413 (84)	621 (93)	
At a birthing center	44 (9.0)	33 (4.9)	
At home/in a private setting	12 (2.4)	16 (2.4)	
Undecided	21 (4.3)		

^a^Pearson's chi-squared test; Fisher's exact test.

As might be expected when recruiting through Facebook and Instagram, the reported rate of weekly social media use was very high (94.5%). However, use of other digital media and tools (such as Instant Messenger, video or audio streaming, and navigation) was comparable to representative samples from Germany and to Swiss census data (see Supplemental Table S2a).^40,41^

### Information sources

**Figure 1.**
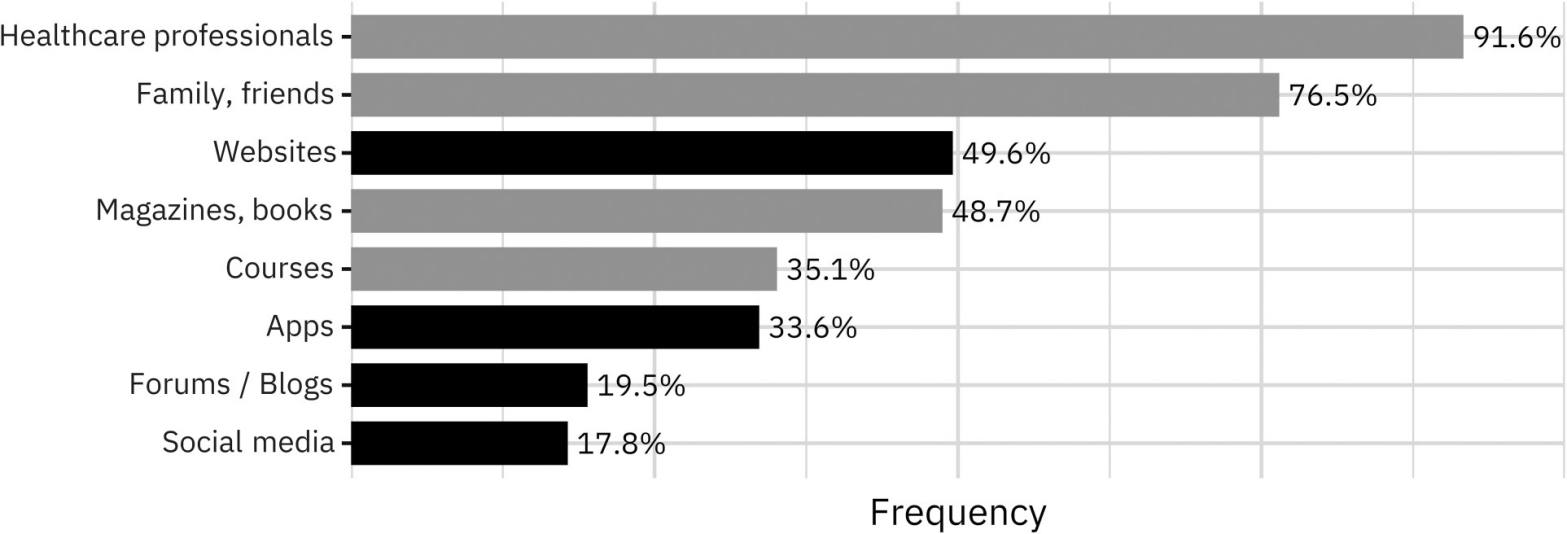
Percentage of women in the perinatal period citing analog (gray) or digital (black) information sources as relevant.

When asked about useful information sources or tools related to pregnancy or motherhood, women most often mentioned healthcare professionals (91.6%) and private networks (76.5%) (Figure 1; Supplemental Table S2b). Other sources (such as books, courses, and apps) were considered more relevant by pregnant women (adj. β = .19, p = .002), primiparous women (adj. β = .25, *p* < .001), and women with a university degree (adj. β = .25, *p* < .001); these sources were consulted less often by women living in intermediate (crude β = −.21, *p* = .005) or rural regions (crude β = −.18, *p* = .01), although the latter effect disappeared in the multivariable regression.

When compared to women living in urban areas, women in rural regions also reported lower self-rated online health information-seeking behavior (adj. β = −.22, *p* = .003). A university degree was a positive predictor of online health information-seeking behavior (adj. β = .24, *p* < .001), as was high-risk pregnancy (adj. β = .13, *p* = .050).

In summary, while healthcare professionals and private networks were the most frequently cited information sources for pregnancy and motherhood, respondents’ use of books, courses, and apps varied significantly by typology (urban/intermediate/rural), educational background, and being primiparous. University-educated women were more inclined to use these latter sources and exhibited more positive attitudes to online health information seeking.

### App usage

The data regarding the most frequently used health apps and websites confirmed the emergence of clear market leaders. The most popular apps were “Pregnancy+” and “Baby+” (Philips Digital UK), which were mentioned by 56.7% and 22.2%, respectively, of the 374 women who provided at least one app name. Other apps like “BabyCenter” (BabyCenter LLC) were mentioned by 14.7% or fewer. Among websites, “Swissmom” was the clear market leader for 89.1% of 488 women who mentioned using at least one website. For other websites, market share was 10.5% or lower.

When asked about their reasons for using apps related to pregnancy or motherhood, most women (52.1%) said they wanted information before making an important decision or wanted to better understand the information provided by healthcare professionals (47.5%) (see Supplemental Table S2c). These reasons regarding health-related information to use apps were less important to women living in rural regions (crude β = −.21, *p* = .014), although this effect ceased to be significant when other variables were taken into account (adj. β = −.16, *p* = .072). Postpartum women (as opposed to pregnant women) (adj. β = .16, *p* = .024), women with pregnancy-related risks (adj. β = .18, *p* = .023), and women with a university degree (β = .20, *p* = .005) were more likely to use apps to inform their decisions or to better understand information provided by healthcare professionals. Social reasons for using apps, such as exchange with other pregnant women or mothers (24.0%) or family and friends (15.3%), were mentioned less often but were more relevant for women with no university degree (adj. β = .17, *p* = .022).

In summary, the primary motivations for using pregnancy or motherhood apps were information gathering for decision-making and clarifying healthcare professionals’ advice, with some notable differences related to education level and pregnancy risk and less emphasis on social exchange.

### Patient data

Women were also asked whether and for what reason they collected health-related data using apps or websites. Respondents most often collected developmental data (e.g. baby movements) (30.9%), data on physical changes (e.g. increases in body weight) (26.8%) and illness symptoms (e.g. nausea) (14.7%) (see Supplemental Table S2d). Only 3% or fewer mentioned collecting data on medical issues such as blood pressure or blood sugar. Just over half (52.1%) reported that they collected no data. In total, 40.3% of all women reported having a maternity record, and a further 3.4% reported having a digital maternity record. As might be expected, health-related data were more often collected by women with pregnancy-related risks (adj. β = .16, *p* = .021), as well as by pregnant women (adj. β = .12, *p* = .040).

### Informational needs

Consistent with these findings, when asked about their informational needs, *pregnant* women showed a heightened interest in topics related to “normal” development and women's behavior, such as the pregnancy timeline (92.9%), pregnancy symptoms (66.5%), medications and vaccinations (39.2%), and nutrition or sport (37.8%) (see Supplemental Table S2e). Topics related to medical issues such as high-risk pregnancy symptoms and therapy (15.7%) or ultrasound and diagnostic testing (10.4%) were mentioned less often.

*Postpartum women* were most interested in normal child development (81.2%), infant care (57.2%), breastfeeding (55.8%), and postpartum recovery (40.0%) (see Supplemental Table S2f). Issues around postpartum depression (16.3%) and healing of perineal wounds or bruises (12.4%) were mentioned less often.

In summary, both pregnant and postpartum women showed a higher interest in “normal” developmental topics and self-care, with less emphasis on medical or risk-related issues.

### App functionalities

Women were also asked about preferred features of a hypothetical health app. Most of the proposed app functionalities were rated positively (see Supplemental Table S2g); these included access to all health-related data (73.4%), prescriptions for medications and orders (72.6%), scheduling appointments with healthcare professionals (71.2%), and automated data exchange with professionals (66.4%). Postpartum women (as opposed to pregnant women) displayed increased interest in functionalities that facilitated health-related data storage and exchange with HCPs (adj. β = .15, *p* = .012). Postpartum women (adj. β = −.29, *p* < .001) and women with a university degree (adj. β = −.26, *p* < .001) were less interested in functionalities that would encourage healthy behaviors (e.g. related to food or exercise (adj. β = −.22, *p* < .001).

Finally, when asked which provider of this hypothetical eHealth app they would consider most trustworthy, around two-thirds of respondents indicated a preference for solutions offered by hospitals (36.3%) or government entities (federal, cantonal; 28.9%) (see Table S2h). Conversely, apps provided by health insurance companies (10.0%) and universities (11.7%) were perceived as less trustworthy.

### Attitudes

In line with their preferences for app functionalities, many women acknowledged the general *advantages* of healthcare digitalization, emphasizing easy access to and overview of personal health data (73.4%), as well as seamless information exchange with HCPs (72.7%). Eliminating unnecessary travel was seen as a significant benefit by 45.6% of respondents. On the other hand, the most commonly cited reasons *against* digitalization were impersonal interaction with healthcare providers (71.0%) and insufficient protection of health data (55.1%) (see Supplemental Table S2h).

Overall, positive attitudes to healthcare digitalization were more frequently seen among women with a university degree (adj. β = .16, *p* = .012) and those with high-risk pregnancies (adj. β = .17, *p* = .010), and consistently, negative attitudes were associated with women without pregnancy-related risks (adj. β = .13, *p* = .048). The specific concern about insufficient protection of health-related data was more important to women with a university degree (adj. β = .16, *p* = .010) and less important to women living in intermediate regions (crude β = −.16, *p* = .036). We also used logistic regression to determine whether *reducing unnecessary travel* was a factor in favoring healthcare digitalization. Interestingly, typology was not a significant predictor in this context (*p*s ≥ .27), but postpartum women (as opposed to pregnant women) were more likely to agree with this argument (adj. OR = 1.39, *p* = .008), as were women with a university degree (adj. OR = 1.29, *p* = .043).

In summary, women who favored healthcare digitalization generally cited convenient access to health data and improved communication with healthcare providers. Positive attitudes were linked to higher education and high-risk pregnancies, although there were notable concerns about impersonal interactions and data security. Contrary to our expectations, women in rural areas did not regard eliminating unnecessary travel as a significant reason for the digitalization of healthcare services.

## Discussion

### Principal findings

Despite its high quality, Switzerland's healthcare system struggles with fragmentation due to its federalist structure and delayed digitalization. This fragmentation is even more pronounced in obstetrics, where women's care over the entire perinatal period is typically delivered by multiple healthcare providers. This leads to inefficient data transfers between institutions and HCPs, an increased risk of incomplete patient records, and extra effort required by HCPs. As healthcare professionals tend to exhibit greater reservations about employing digital tools, we sought to understand whether pregnant and postpartum women could potentially drive increasing digitalization, especially as a means of bridging gaps in the flow of information. To that end, the present study sought to clarify women's use of digital tools and their needs and attitudes in this regard, focusing in particular on a comparison of rural and urban women's feelings about telemedicine.

In this sample of perinatal women recruited through social media, healthcare professionals, family, and personal networks were the primary sources of information about concerns related to pregnancy or motherhood. While many participants also used apps and websites (and some even collected data themselves), these rarely related to medical issues or health-related data. Participants most often used apps such as “Pregnancy+” and “Baby+” (Philips Digital UK), which provide daily or weekly updates on physiological development and enable women to track their weight or their baby's growth. A clear majority expressed a preference for using an app to access their data and prescriptions and to schedule appointments, and this finding aligns with other Swiss surveys of the general population.^8^ Conversely, one significant concern about healthcare digitalization was the impersonal nature of interactions with professionals.

About 40% of participants reported having a maternity record, and an additional 3.4% had a digital maternity record. The Swiss maternity record (“Mutterpass”) offers pregnant women a comprehensive summary of relevant medical information, examinations, and procedures throughout their pregnancy (cf. Federal Statistical Office^42^ and Koch^43^). However, there is no official maternity record, and only a few institutions offer such records in digital form, which makes it more difficult to share data,^44^ not least because midwives and physicians use paper-based maternity records of various kinds. Maternity records could support minimal data exchange between health providers, but to be of use for seamless data transfer, these documents should be generally available in digital form. The present findings suggest that most women would be interested in having access to all relevant data in the prepartum and postpartum periods.

### Typology, stage, pregnancy risks, and university education

Despite their exploratory nature, our analyses yielded some clear insights into the factors underlying healthcare app utilization and attitudes to eHealth and healthcare digitalization. For our respondents, healthcare professionals were the primary source of information about health-related issues. Women living in *intermediate or rural areas* were less likely to consult other sources of information (such as books and courses) or to use websites or apps to understand or address health-related issues. In particular, for pregnant women and women living in rural areas, their strong reliance on healthcare professionals, family, and friends largely explains why the elimination of unnecessary travel is not a compelling argument for healthcare digitalization. In the context of the COVID-19 pandemic, one German study of midwives and mothers identified reduced travel time as an argument in favor of digital care.^45^ However, it is important to note that travel times in Switzerland are relatively short, as 99.8% of the Swiss population can reach a general hospital in less than half an hour by car.^46^

Crucially, any differences between urban and rural women largely disappeared in multivariate analyses that included *university education*. Women with a university degree exhibited more positive attitudes to online health information-seeking and were more likely to consult additional sources, including apps, to inform health-related decisions. Notably, compared to women living in rural regions, they were also more open to the argument that healthcare digitalization could minimize unnecessary travel for medical purposes. Interestingly, despite their more positive attitude to digitalization, they were also more alert to potential data protection issues.

The association between attitudes to online information-seeking behavior and educational status can probably be explained in terms of eHealth literacy (cf. Wong and Cheung^47^), which again seems related to having a university degree (e.g. ^48^^–50^), although not all studies agree (cf. ^51^). Less educated women may be less accustomed to consulting digital sources for health-related matters, and low self-efficacy in this area may increase the challenges of interpreting and critically evaluating any such information.^52,53^ While our results suggest that the negative association between rurality and eHealth literacy is driven by differences in education, evidence of any relationship between *rurality* and eHealth literacy remains limited.^54^

Differences in education could also explain why women in this survey used apps and websites, but not for medical issues such as postpartum depression or wound healing. Women use digital tools for various reasons—e.g. information about physiological changes in their own or their baby's body—but they consult HCPs for medical issues.^55^ HCPs are viewed as credible, whereas the quality of information from the internet is often questioned.^55,56^ Women find it difficult to assess the quality of this information and make decisions based on it.^57^ This means that they are required to take on the role of “lay researchers” who have to compare and evaluate information from different sources and channels.^56,58^ This likely explains why women with a lower level of education feel insecure about this critical aspect of health literacy.

Compared to *postpartum* and *multiparous* women, respectively, *pregnant* and *nulliparous* women exhibited a greater overall need for information, which manifested in their more frequent use of both app-based and traditional health information sources. More specifically, pregnant women more often reported using pregnancy-related apps but did not use apps for other health-related purposes. Conversely, postpartum women (as opposed to pregnant women) seemed more interested in apps that would help them to better understand the information provided by HCPs and favored apps that would facilitate appointment scheduling and video calls with their HCP or automated data exchange. Postpartum women were also consistently more open to the argument that such applications could eliminate unnecessary travel.

The study confirms that *first-time mothers* exhibit a high demand for information and consume more courses, books, and apps than their more experienced counterparts. *Pregnant* women tend to prioritize in-person contact to ensure their child's well-being, placing less emphasis on digital solutions. On the other hand, *mothers—*especially working mothers—may have tighter schedules and are therefore more likely to use apps for medical purposes and to value solutions that save travel time. As mentioned earlier, Pflugeisen and Mou^19^ found that, for subsequent pregnancies, women favor virtual care combining face-to-face visits with videoconferencing rather than traditional models of care.

Finally, as might be expected, *women facing pregnancy risks* were much more interested in using apps for health-related purposes—for instance, to make it easier to collect data and monitor risks. Overall, these women displayed more positive attitudes to healthcare digitalization. A substantial array of applications already enables pregnant women to monitor various health parameters for physical training and weight management^21^, e.g. ^59–62^ Our findings suggest that the growing number of women with specific health risks and multicomorbidities (cf. ^63–65^) are most likely to engage with and benefit from digital monitoring tools for collecting their own health-related data.^66^ However, our focus has been primarily on medical risks, while mental health issues, which are more prevalent and often stigmatized,^59^ present an equally significant area where digital intervention could offer substantial benefits.^60,61^

### Implications

The present findings clearly indicate that professionals are the primary source of healthcare information for our target population, and this tends to be even truer of women living in rural areas. In light of the increasing shift towards outpatient services, it seems unlikely that telemedicine applications will significantly impact core obstetric consultations, particularly during pregnancy. Instead, the focus should be on enhanced patient management, remote monitoring, patient-generated data, and data transfer with healthcare professionals, prioritizing the postpartum period rather than pregnancy. Given women's interest in pregnancy and fetal development and the promotion of healthy behaviors, applications should ideally use these “lifestyle” issues as stepping stones to introduce other functionalities that facilitate communication and data transfer with healthcare professionals. Finally, women's interest in accessing their own health-related data underlines the potential of a digital maternity record to bridge gaps in the flow of information between HCPs and institutions by providing a more integrated and accessible way of managing health data across the entire perinatal period.

Given some women's challenges in assessing the quality of websites and apps, and their reluctance to use this information for medical purposes, HCPs should guide women in the perinatal period toward trustworthy sources,^55^ introduce specific digital tools they find helpful^67^ or direct them to safe digital communities.^68^ Birth preparation courses could also promote digital self-help by helping women identify trustworthy digital sources and manage the information they find. Due to limited capacity among healthcare professionals, Massive Open Online Courses also serve as a means to enhance digital health literacy.^69^

Most of the women in the present study also expressed a preference for trustworthy apps provided by hospitals or government bodies (cf. Meldgaard et al.^57^). While concerns persist about information quality when market interests predominate,^16,70^ it is also crucial to note that many apps address only a limited spectrum of physiological development issues. This narrow focus seems insufficient to foster empowerment, especially in relation to health-related matters and understanding the decisions made by healthcare professionals. The use of technology should empower women to have more control and greater participation in health-related decisions, fostering independence and enabling them to be “active participant in their health.”^55,56,71^ However, this empowerment is unattainable if digital tools do not align with women's existing knowledge and skills.^68^ For instance, many mHealth applications are designed for a well-educated ideal user.^71^ Additionally, Birati et al.^72^ noted that only a minority of app- and web-based systems for pregnant women with gestational diabetes mellitus consider language and cultural diversity, despite the crucial role of different cultures and religions in establishing new habits.

Addressing these challenges requires a codesign approach where perinatal women, HCPs and developers collaborate from the start. First, materials and tools must either match the respective levels of health and digital literacy or utilize simple language and simplified navigation.^72^ Second, content should be as personalized and culturally tailored as possible to meet specific needs, patient journeys, and cultural backgrounds for effectiveness.^68,73^ Also, tracking behaviors and data entry become more engaging if systems provide timely feedback suited to the specific circumstances of the woman, for instance their current stage in pregnancy (“Just-in-Time Adaptive Interventions” (JITAIs), cf. Lee et al.^68^, Nahum-Shani et al.^74^). Third, for digital health interventions to be relevant regarding medical decisions, they must be localized and adapted to the specific health services available in the woman's region.^68^ Therefore, the collaborative development approach is most likely to ensure effective digital tools, even though the overall effort might be greater. Additionally, app development that positions the professional as central coordinator while also taking account of the user perspective seems optimal for service improvement. This would also ensure thorough evaluation of whether the promise of empowerment can indeed be fulfilled beyond monitoring pregnancy-related risks or promoting healthy behaviors.^75,76^ Similarly, while concerns about data privacy and security are not that common among women or patients in general, many current apps seem poor in terms of privacy,^77^ and there must be a more concerted effort to develop standards and regulations that define minimal requirements.

### Strengths and limitations

The present study has some significant limitations that may affect the interpretation of our results. As in any survey with nonrandom sampling, self-selection bias is most likely an issue in the current study, although to an unknown degree. Additionally, recruiting participants through social media is likely to limit the representativeness of our sample. However, it was comparable to census data, and many of our results align with other studies. The exclusively German-language questionnaire represents another significant limitation. Switzerland has four national languages, primarily spoken in their respective regions. German is spoken by around two-thirds of the country's population. Therefore, our study results cannot be generalized to the entire Swiss population, especially not to first-generation foreigners, only a third of whom speak German. The generalizability of these findings to other countries is also limited, especially with regard to the urban–rural typology, as the relevant travel distances are notably shorter in Switzerland. Importantly, most of the questionnaire items were developed specifically for the purposes of the present study and were not rigorously validated. Nevertheless, the internal consistency of our results and the alignment with existing research lend support to our choice of methodology.

## Conclusion

The present study underscores the need for digital tools that not only provide information on physiological developments but also empower women by facilitating communication and data transfer with healthcare professionals, especially in the postpartum period. Our results highlight the importance of developing trustworthy user-focused digital applications that prioritize women's empowerment and data privacy. This in turn suggests the need for a shift in the current approach to healthcare digitalization, placing special emphasis on bridging gaps in the flow of information and promoting a more inclusive and effective healthcare system.

## Supplemental Material

sj-docx-1-dhj-10.1177_20552076241277671 - Supplemental material for Digital health in perinatal care: Exploring usage, attitudes, and needs among Swiss women in urban and rural settings Supplemental material, sj-docx-1-dhj-10.1177_20552076241277671 for Digital health in perinatal care: Exploring usage, attitudes, and needs among Swiss women in urban and rural settings by Stephan Oelhafen in DIGITAL HEALTH

sj-docx-2-dhj-10.1177_20552076241277671 - Supplemental material for Digital health in perinatal care: Exploring usage, attitudes, and needs among Swiss women in urban and rural settings Supplemental material, sj-docx-2-dhj-10.1177_20552076241277671 for Digital health in perinatal care: Exploring usage, attitudes, and needs among Swiss women in urban and rural settings by Stephan Oelhafen in DIGITAL HEALTH

## Notes

a. In Switzerland, a canton is an administrative subdivision, similar to a state in other countries.
