# Supplementary material for: Digital health in perinatal care: Exploring usage, attitudes, and needs among Swiss women in urban and rural settings
Source: Digit Health. 2024 Sep 2;10:20552076241277671. doi: 10.1177/20552076241277671 (PMC11372771; doi:10.1177/20552076241277671)
Supplement: sj-docx-1-dhj-10.1177_20552076241277671 - Supplemental material for Digital health in perinatal care: Exploring usage, attitudes, and needs among Swiss women in urban and rural settings [file sj-docx-1-dhj-10.1177_20552076241277671.docx]

Additional File 1

[Table S1a. List of independent variables, item wording (English). 2](#_Toc160957286)

[Table S1b. List of independent variables, item wording (German). 9](#_Toc160957287)

Note: English items were not used in the current study and were only translated for the purpose of comprehensibility.

# **Table S1a. List of independent variables, item wording (English).**

| **Variable** | **Question** | **Response options / scale** |
| --- | --- | --- |
| Pregnancy_Status | Are you currently pregnant or have you given birth in the last 12 months? | - Yes, I am currently pregnant. - Yes, I have given birth in the last 12 months. - No |
| Gestational age | How many weeks pregnant are you? | - ______ |
| Nulliparous | Will this be your first birth? | - Yes, this will be my first birth. - No |
| Risk pregnancy | During this pregnancy, have you needed any treatment or monitoring due to complications, health issues, or psychological concerns? Select all that apply. | - No - Yes, outpatient treatment - Yes, hospitalized (with an overnight stay) - Other measures: ______ |
| Main caregiver pregnancy | Who is your primary medical contact during this pregnancy? | - General Practitioner - Obstetrician/Gynecologist - Midwife - Other: ______ |
| Planned place of birth | Where do you plan to give birth? | - In a hospital - At a birthing center - At home / in a private setting - Undecided |
| Birth mode preference | How do you wish to deliver your child? | - Vaginally - By cesarean section (on medical advice) - By cesarean section (by personal choice) |
| Child’s age | How many months ago did you give birth? | - ______ |
| Nulliparous | Was this your first birth? | - Yes, it was my first birth. - No |
| Risk pregnancy | During your pregnancy, did you require treatment or monitoring due to complications, health issues, or psychological concerns? Please check all that apply. | - No - Yes, outpatient treatment - Yes, hospitalized (with an overnight stay) - Other measures: ______ |
| Main caregiver pregnancy | Who was your primary medical contact during this pregnancy? | - General Practitioner - Obstetrician/Gynecologist - Midwife - Other: ______ |
| Place of birth | Where did you give birth? | - In a hospital - At a birthing center - At home / in a private setting |
| Birth mode | How did you deliver your child? | - Vaginally - Vaginally with the assistance of vacuum extraction/forceps - Planned cesarean section (on medical advice) - Planned cesarean section (by personal choice) - Unplanned cesarean section |
| Digital services | Which digital services do you use once or several times per week? | - Online videos (e.g. YouTube) - Navigation (e.g. Google Maps) - Instant messaging (e.g. WhatsApp, Signal, SMS) - Social media (e.g. Facebook, Instagram, Twitter) - Online shopping - Video calls (e.g. WhatsApp, Zoom, FaceTime, Skype) - Voice control (e.g. Siri, Alexa, Google Assistant) - Streaming services (e.g. Spotify, Netflix) - Health/Fitness apps |
| Health information seeking online (cf. [26]) | To what extent do the following statements apply to you?   - I use the internet when I am unsure if a professional has given me correct information. - When I have to make a decision regarding my health, I seek information on the internet. - The internet can be useful when it's unclear whether to see a doctor about certain symptoms. | (Likert scale)   - Strongly Disagree - Disagree - Neither Agree nor Disagree - Agree - Strongly Agree |
| Information sources | What are useful sources of information or resources for you regarding [pregnancy and childbirth/the postpartum period and motherhood]?  Please check all that apply. | - Healthcare professionals - Family, friends - Magazines, books - Social media - Websites - Apps - Forums / Blogs - Courses |
| Apps | Which apps related to [pregnancy and childbirth/the postpartum period and motherhood] do you use most frequently? | - App #1: ____________ - App #2: ____________ - App #3: ____________ |
| Blogs | Which forums or blogs on the topics of [pregnancy and childbirth/the postnatal period and motherhood] do you frequent most often? | - Forum/Blog #1: ____________ - Forum/Blog #2: ____________ - Forum/Blog #3: ____________ |
| Websites | Which websites on the topics of [pregnancy and childbirth/the postnatal period and motherhood] do you frequent most often? | - Website #1: ____________ - Website #2: ____________ - Website #3: ____________ |
| App reasons | Why do you use the internet or apps for topics related to [pregnancy and childbirth/the postnatal period and motherhood]?  Please check all that apply. | - To prepare for discussions with healthcare professionals - To better understand information provided by professionals - To gather information before making important decisions - To keep friends and family updated - To interact with other [pregnant women/mothers] - I do not use apps or the internet for these purposes - Other reasons: ____________ |
| Data collection | Do you use apps or websites to track the following health data? Please check all that apply. | - Illness symptoms (e.g. nausea, mood swings) - Physical changes (e.g. increase in belly size, body weight) - Baby's development (e.g. fetal movements or heartbeats) - Blood pressure - Blood sugar - Protein in urine - No, I do not track any data. - Other data: ____________ |
| Maternity record | Do you have a maternity record? | - Yes, a paper maternity record - Yes, a digital maternity record (App or USB stick) - No |
| Maternity record (name) | Which maternity record do you use? (Please specify the name, publisher, or a description) | - ____________ |
| Pregnancy topics | Which topics do you search online or with apps regarding pregnancy? Please check all that apply. | - High-risk pregnancy: Symptoms & Treatment - Results of ultrasound / diagnostic tests - Twins or multiple births - Nutrition & Exercise - Sleep - Sexuality - Permitted medications, vaccinations - Consumption of coffee, nicotine, or alcohol - Birthplace - Pregnancy symptoms - What happens in each week of pregnancy - I do not research online or with apps - Other topics: ____________ |
| Postpartum topics | Which topics do you search online or with apps regarding the postpartum period? Please check all that apply. | - Breastfeeding, mastitis - Infant care, development - Postnatal recovery - Healing of perineal wounds, bruises - Nutrition & Exercise - Sleep - Sexuality - Urinary incontinence - Postpartum depression - Consumption of coffee, nicotine, or alcohol - Child's development - I do not search online or with apps - Other topics: ____________ |
| App features preference | Imagine a new health app or website for [pregnant women/mothers] combining several features. How useful would the following features be to you?   - All health data in one app - Appointment scheduling with healthcare professionals - Video consultation with a healthcare professional - Prescriptions for medications/orders in the app - Reminders for health-promoting behaviors (e.g. for eating, exercise) - Tracking my health data (e.g. blood pressure) - Automatic sharing of data with healthcare professionals | (Likert scale)   - Not at all useful - Not very useful - Neutral - Somewhat useful - Very useful |
| App features other | Are there any other features of such an app that would be particularly useful for you? | - ____________ |
| Digitalization advantages | What advantages do you see in the increasing use of digital technologies in healthcare for communication, and the storage and transfer of data? This includes electronic health records or video consultations. Please check all that apply. | - Simplified information exchange - Avoiding unnecessary travel to healthcare professionals - Reduced risk of on-site infection (e.g. COVID-19) - Faster access and overview of personal health data - Monitoring my health status at home - No advantages - Other: ____________ |
| Digitalization disadvantages | What disadvantages do you see in the increasing use of digital technologies in healthcare? Please check all that apply. | - Poor internet connection or technical issues - Insufficient protection of my health data - Impersonal contact with healthcare professionals - Difficulty in reaching healthcare professionals - Having to collect data myself - Being at the mercy of computer decisions - No disadvantages - Other: ____________ |
| Digital health provider preference | Which provider of digital health technologies would you trust the most? | - Private provider - Health insurance company - Federal / State government - University / College - Professional associations - Hospital |
| Postal code | What is your postal code? (This information is only used to associate your residence with a region.) | - ______ |
| Drive | Approximately how long is the drive from your home to the nearest hospital? (even if you do not own a car) | - ______ |
| Household (pregnancy) | Who do you live with in the same household? Please check all that apply. | - Spouse / Partner - Parents or parent - Other adult person(s) - School-aged children - Pre-school children - I currently live alone |
| Household (postpartum) | Who do you live with in the same household? Please check all that apply. | - Spouse / Partner - Parents or parent - Other adult person(s) - School-aged children - Non-school-aged children (excluding the newborn) - I live alone with the newborn |
| Mother’s age | How old are you? | - under 20 - 20-24 - 25-29 - 30-34 - 35-39 - 40-44 - 45 or older |
| Marital status | What is your marital status? | - Married / registered partnership - Single - Divorced / widowed / other |
| Professional status | What is your current employment situation? Please check all that apply. | - Employed full-time (80-100%) - Employed part-time (<80%) - Working in a family business - In education/training - Homemaker - Self-employed - Unemployed - Receiving social assistance / disability benefits - Other: __________________ |
| Education | What is the highest level of school or training you have completed? | - Compulsory education - Apprenticeship - Grammar school, Vocational A level, specialized secondary school certificate (FMS), Vocational college (DMS) - Higher technical and vocational training - University of Applied Sciences, educational college - University, Federal Institute of Technology (EPFL, ETH) - Other: __________________ |
| Health insurance | How are you insured for health care? | - General coverage - Semi-private - Private - I don’t know |

# **Table S1b. List of independent variables, item wording (German).**

| **Variable** | **Question** | **Response options / scale** |
| --- | --- | --- |
| Pregnancy_Status | Sind Sie schwanger oder haben Sie in den letzten 12 Monaten  geboren? | - Ja, ich bin aktuell schwanger. - Ja, ich habe in den letzten 12 Monaten geboren. - Nein |
| Gestational age | In welcher Schwangerschaftswoche sind Sie? | - ______ |
| Nulliparous | Wird dies Ihre erste Geburt sein? | - Ja, das wird meine erste Geburt sein. - Nein |
| Risk pregnancy | Müssen oder mussten Sie in der aktuellen Schwangerschaft aufgrund  von Komplikationen, gesundheitlichen oder psychischen Problemen  behandelt oder Ihr Zustand überwacht werden? Es sind mehrere Antworten möglich. | - Nein - Ja, ambulant - Ja, stationär im Spital (mit Übernachtung) - Andere Massnahmen: ______ |
| Main caregiver pregnancy | Wer ist in dieser Schwangerschaft Ihre erste medizinische  Ansprechperson? | - Hausärztin/-arzt - Gynäkolog/in - Hebamme - andere: ______ |
| Planned place of birth | Wo ist die Geburt geplant? | - im Spital - im Geburtshaus - zuhause / in privater Umgebung - ist noch offen |
| Birth mode preference | Auf welchem Weg möchten Sie Ihr Kind gebären? | - vaginal - per Kaiserschnitt (auf Anraten einer medizinischen Fachperson) - per Kaiserschnitt (auf eigenen Wunsch) |
| Child’s age | Vor wie vielen Monaten haben Sie geboren? | - ______ |
| Nulliparous | War dies Ihre erste Geburt? | - Ja, das war meine erste Geburt. - Nein |
| Risk pregnancy | Mussten Sie in der Schwangerschaft aufgrund von Komplikationen,  gesundheitlichen oder psychischen Problemen behandelt oder Ihr  Zustand überwacht werden? Es sind mehrere Antworten möglich. | - Nein - Ja, ambulant - Ja, stationär im Spital (mit Übernachtung) - Andere Massnahmen: ______ |
| Main caregiver pregnancy | Wer war in dieser Schwangerschaft Ihre erste medizinische  Ansprechperson? | - Hausärztin/-arzt - Gynäkolog/in - Hebamme - andere: ______ |
| Place of birth | Wo fand diese Geburt statt? | - im Spital - im Geburtshaus - zuhause / in privater Umgebung |
| Birth mode | Auf welchem Weg haben Sie Ihr Kind geboren? | - vaginal - vaginal mit Vakuum/Saugglocke/Geburtszange - geplanter Kaiserschnitt (auf Anraten einer medizinischen Fachperson) - geplanter Kaiserschnitt (auf eigenen Wunsch) - ungeplanter Kaiserschnitt |
| Digital tools (general) | Welche digitalen Dienste verwenden Sie ein- oder mehrmals pro  Woche? | - Online-Videos (z.B. Youtube) - Navigation (z.B. Google Maps) - Instant-Messaging (z.B. WhatsApp, Signal, SMS) - Social Media (z.B. Facebook, Instagram, Twitter) - Online-Shopping - Videotelefonie (z.B. WhatsApp, Zoom, Facetime, Skype) - Sprachsteuerung (z.B. Siri, Alexa, Google Assistant) - Streaming (z.B. Spotify, Netflix) - Gesundheits-/Fitnessapps |
| Health information seeking online | Inwiefern treffen folgende Aussagen auf Sie zu?   - Ich nutze das Internet, wenn ich unsicher bin, ob mich eine Fachperson korrekt informiert hat. - Wenn ich eine Entscheidung fällen muss, die meine Gesundheit betrifft, informiere ich mich über das Internet. - Wenn unklar ist, ob man mit gewissen Symptomen zum Arzt gehen sollte, kann das Internet nützlich sein. | (Likert scale)   - trifft überhaupt nicht zu - trifft eher nicht zu - weder noch - trifft eher zu - trifft voll und ganz zu |
| Information sources | Welches sind für Sie bezüglich [Schwangerschaft und  Geburt/Wochenbett und Mutterschaft] nützliche Informationsquellen oder Hilfsmittel? Es sind mehrere Antworten möglich. | - Fachpersonen - Familie, Freundinnen, Freunde - Zeitschriften, Bücher - Soziale Medien - Webseiten - Apps - Foren / Blogs - Kurse |
| Apps | Welche Apps zu den Themen [Schwangerschaft und  Geburt/Wochenbett und Mutterschaft] benutzen Sie am häufigsten? | - App #1: ____________ - App #2: ____________ - App #3: ____________ |
| Blogs | Welche Foren oder Blogs zu den Themen [Schwangerschaft und  Geburt/Wochenbett und Mutterschaft] besuchen Sie am häufigsten? | - Forum/Blog #1: ____________ - Forum/Blog #2: ____________ - Forum/Blog #3: ____________ |
| Websites | Welche Webseiten zu den Themen [Schwangerschaft und  Geburt/Wochenbett und Mutterschaft] nutzen Sie am häufigsten? | - Webseite #1: ____________ - Webseite #2: ____________ - Webseite #3: ____________ |
| App reasons | Weshalb verwenden Sie das Internet oder Apps rund um die Themen [Schwangerschaft und  Geburt/Wochenbett und Mutterschaft]? Kreuzen Sie alle an, die zutreffen. | - bereite mich auf Gespräch mit Fachperson vor - möchte Informationen durch Fachperson besser verstehen - sammle Informationen vor einer wichtigen Entscheidung - halte Freunde und Familie auf dem Laufenden - tausche mich mit anderen [Schwangeren/Müttern] aus - verwende keine Apps oder das Internet für diesen Zweck - andere Gründe: ____________ |
| Data collection | Verwenden Sie Apps oder Webseiten, um folgende Gesundheitsdaten  zu sammeln? Kreuzen Sie alle an, die zutreffen. | - Krankheitssymptome (z.B. Übelkeit, Gemütsschwankungen) - körperliche Veränderungen (z.B. Zunahme des Bauchumfangs, des Körpergewichts) - Entwicklung des Babys (z.B. Kindsbewegungen oder Herztöne) - Blutdruck - Blutzucker - Eiweissausscheidung im Urin - Nein, ich sammle keine Daten. - andere Daten: ____________ |
| Maternity record | Besitzen Sie einen Mutterpass? | - ja, einen Mutterpass auf Papier - ja, einen digitalen Mutterpass (App oder USB-Stick) - nein |
| Maternity record (name) | Welchen Mutterpass verwenden Sie? (Name, Herausgeber oder Umschreibung) | - ____________ |
| Pregnancy topics | Zu welchen Themen informieren Sie sich online oder mithilfe von  Apps? Kreuzen Sie alle Antworten an, die zutreffen. | - Risikoschwangerschaft: Symptome & Therapie - Resultate des Ultraschalls / von diagnostischen Tests - Mehrlinge - Ernährung & Sport - Schlaf - Sexualität - erlaubte Medikamente, Impfungen - Kaffee-, Nikotin- oder Alkoholkonsum - Geburtsort - Schwangerschaftsbeschwerden - was in welcher Schwangerschaftswoche passiert - ich informiere mich nicht online oder mithilfe von Apps - andere Themen: ____________ |
| Postpartum topics | Zu welchen Themen informieren Sie sich online oder mithilfe von  Apps? Kreuzen Sie alle Antworten an, die zutreffen. | - Stillen, Brustentzündung - Säuglingspflege, Entwicklung - Rückbildung - Wundheilung Damm, Blutergüsse - Ernährung & Sport - Schlaf - Sexualität - Blasenschwäche - Wochenbettdepression - Kaffee-, Nikotin- oder Alkoholkonsum - Entwicklung des Kindes - ich informiere mich nicht online oder mithilfe von Apps - andere Themen: ____________ |
| App features preference | Stellen Sie sich vor, eine neue Gesundheits-App oder -Webseite für  [Schwangere/ Mütter] würde mehrere Funktionen vereinen. Wie nützlich wären für Sie die unten aufgelisteten  Funktionen?   - Alle Gesundheitsdaten in einer App - Terminvereinbarung mit Fachpersonen - Videokonsultation mit Fachperson - Rezepte für Medikamente / Verordnungen in App - Erinnerungsfunktion für gesundheitsförderndes Verhalten (z.B. für Essen, - Erfassen meiner Gesundheitsdaten (z.B. Blutdruck) - Automatischer Austausch der Daten mit Fachperson. | Likert scale:   - gar nicht nützlich - eher nicht nützlich - weder noch - eher nützlich - sehr nützlich |
| App features other | Gäbe es andere Funktionen einer solchen App, die für Sie besonders nützlich wären? | - ____________ |
| Digitalization advantages | Welche Vorteile bestehen aus Ihrer Sicht, wenn im Gesundheitswesen  zunehmende digitale Technologien für die Kommunikation und die  Speicherung und Übertragung von Daten eingesetzt werden? Dazu  gehören z.B. das elektronische Patientendossier oder Konsultationen per Videotelefonie. Kreuzen Sie alle Antworten an, die zutreffen. | - unkomplizierter Informationsaustausch - Vermeidung unnötiger Anfahrtswege zur Fachperson - Verminderung Ansteckungsrisiko vor Ort (z.B. Covid-19) - schneller Zugriff und Überblick auf eigene Gesundheitsdaten - Überwachung meines Gesundheitszustandes zuhause - keine Vorteile - andere: ____________ |
| Digitalization disadvantages | Welche Nachteile sehen Sie, wenn im Gesundheitswesen zunehmend  digitale Technologien eingesetzt werden? Kreuzen Sie alle Antworten an, die zutreffen. | - schlechte Internetverbindung oder technische Probleme - ungenügender Schutz meiner Gesundheitsdaten - unpersönlicher Kontakt mit Fachperson - erschwerte Erreichbarkeit der Fachperson - selbst Daten erheben zu müssen - den Entscheidungen eines Computers ausgeliefert sein - keine Nachteile - andere: ____________ |
| Digital health provider preference | In welchen Anbieter von digitalen Technologien im Gesundheitswesen  hätten Sie am meisten Vertrauen? | - privater Anbieter - Krankenkasse - Bund / Kantone - Hochschule / Universität - Berufsverbände - Spital |
| Postal code | Wie lautet Ihre Postleitzahl?  Die Angabe wird nur verwendet, um Ihren Wohnort einer Landesregion zuzuordnen | - ______ |
| Drive | Wie lange dauert ungefähr die Fahrt mit dem Auto von Ihrem Wohnort ins nächstgelegene Spital? (auch wenn Sie kein Auto besitzen sollten) | - ______ |
| Household (pregnancy) | Mit wem leben Sie im gleichen Haushalt? Es sind mehrere Antworten möglich. | - Ehemann / Ehefrau / Partner*in - Eltern oder Elternteil - andere erwachsene Person(en) - schulpflichtige Kinder - nicht schulpflichtige Kinder - ich lebe momentan allein |
| Household (postpartum) | Mit wem leben Sie im gleichen Haushalt? Es sind mehrere Antworten möglich. | - Ehemann / Ehefrau / Partner*in - Eltern oder Elternteil - andere erwachsene Person(en) - schulpflichtige Kinder - nicht schulpflichtige Kinder (ohne das Neugeborene) - ich lebe allein mit dem Neugeborenen |
| Mother’s age | Wie alt sind Sie? | - unter 20 - 20-24 - 25-29 - 30-34 - 35-39 - 40-44 - 45 oder älter |
| Marital status | Welchen Zivilstand haben Sie? | - verheiratet / eingetragene Partnerschaft - ledig - geschieden / verwitwet / anderer |
| Professional status | Was ist Ihre aktuelle berufliche Situation? Es sind mehrere Antworten möglich. | - arbeitnehmend, Vollzeit (80-100%) - arbeitnehmend, Teilzeit (<80%) - berufstätig im Familienbetrieb - in Ausbildung - Hausfrau - selbstständig erwerbend - arbeitslos - beziehe Sozialhilfe / IV - andere: __________________ |
| Education | Welche Schule oder Ausbildung haben Sie zuletzt abgeschlossen? | - obligatorische Schule - Berufslehre - Gymnasium, Berufsmatura, FMS, DMS - Höhere Fachs- und Berufsbildung - Fachhochschule, PH - Universität, ETH, EPFL - andere: __________________ |
| Health insurance | Wie sind sie krankenversichert? | - allgemein - halbprivat - privat - Ich weiss es nicht |
