# Supplementary material for: Digital health in perinatal care: Exploring usage, attitudes, and needs among Swiss women in urban and rural settings
Source: Digit Health. 2024 Sep 2;10:20552076241277671. doi: 10.1177/20552076241277671 (PMC11372771; doi:10.1177/20552076241277671)
Supplement: sj-docx-2-dhj-10.1177_20552076241277671 - Supplemental material for Digital health in perinatal care: Exploring usage, attitudes, and needs among Swiss women in urban and rural settings [file sj-docx-2-dhj-10.1177_20552076241277671.docx]

**Table S2a**

*Weekly usage of digital services*

|  | *n* (%) |
| --- | --- |
| Online videos (e.g. YouTube) | 430 (37%) |
| Navigation (e.g. Google Maps) | 587 (51%) |
| Instant messaging (e.g. WhatsApp, Signal, SMS) | 1,057 (91%) |
| Social media (e.g. Facebook, Instagram, Twitter) | 1,096 (94%) |
| Online shopping | 457 (39%) |
| Video calls (e.g. WhatsApp, Zoom, FaceTime, Skype) | 472 (41%) |
| Voice control (e.g. Siri, Alexa, Google Assistant) | 43 (3.7%) |
| Streaming services (e.g. Spotify, Netflix) | 657 (57%) |
| Health/Fitness apps | 208 (18%) |

*Note.* *N* = 1,160

**Table S2b**

*Useful sources of information about pregnancy and childbirth or the postpartum period (Components derived from PCA)*

|  | *n* (%) |
| --- | --- |
| Component 1: HCP vs. online |  |
| Healthcare professionals | 1,063 (92%) |
| Forums/Blogs (R) | 226 (19%) |
| Social media (R) | 207 (18%) |
| Component 2: Other sources |  |
| Family, friends | 887 (76%) |
| Magazines, books | 565 (49%) |
| Websites | 575 (50%) |
| Apps | 390 (34%) |
| Courses | 407 (35%) |

*Note.* *N* = 1,160. Items marked (R) loaded negatively on the respective component of the PCA.

**Table S2c**

*Reasons for using internet or mobile applications related to pregnancy and childbirth or the postpartum period (Components derived from PCA)*

|  | *n* (%) |
| --- | --- |
| Component 1: Health-related information |  |
| To prepare for discussions with healthcare professionals | 275 (33%) |
| To better understand information provided by professionals | 402 (48%) |
| To gather information before making important decisions | 441 (52%) |
| Component 2: Social aspects |  |
| To keep friends and family updated | 130 (15%) |
| To interact with other pregnant women/mothers | 203 (24%) |

*Note.* *N* = 846. This item was shown only to participants who identified websites or mobile apps as relevant sources of information in the previous item.

**Table S2d**

*Types of data tracked via mobile applications or websites*

|  | *n* (%) |
| --- | --- |
| Illness symptoms (e.g. nausea, mood swings) | 171 (15%) |
| Physical changes (e.g. increase in belly size, body weight) | 311 (27%) |
| Baby's development (e.g. foetal movements or heartbeat) | 359 (31%) |
| Blood pressure | 35 (3.0%) |
| Blood sugar | 33 (2.8%) |
| Protein in urine | 11 (0.9%) |

*Note.* *N* = 1,160.

**Table S2e**

*Pregnancy-related topics researched via internet or mobile applications*

|  | *n* (%) |
| --- | --- |
| High-risk pregnancy: symptoms & treatment | 77 (16%) |
| Results of ultrasound/diagnostic tests | 51 (10%) |
| Twins or multiple births | 13 (2.7%) |
| Nutrition & Exercise | 185 (38%) |
| Sleep | 76 (16%) |
| Sexuality | 73 (15%) |
| Permitted medications, vaccinations | 192 (39%) |
| Consumption of coffee, nicotine, or alcohol | 73 (15%) |
| Birthplace | 97 (20%) |
| Pregnancy symptoms | 326 (67%) |
| What happens in each week of pregnancy | 456 (93%) |

*Note.* *N* = 490. This item was shown only to pregnant women.

**Table S2f**

*Postpartum period topics researched via the internet or mobile applications*

|  | *n* (%) |
| --- | --- |
| Breastfeeding, mastitis | 374 (56%) |
| Infant care, development | 383 (57%) |
| Postnatal recovery | 268 (40%) |
| Healing of perineal wounds, bruises | 83 (12%) |
| Nutrition & Exercise | 189 (28%) |
| Sleep | 196 (29%) |
| Sexuality | 103 (15%) |
| Urinary incontinence | 48 (7.2%) |
| Postpartum depression | 109 (16%) |
| Consumption of coffee, nicotine, or alcohol | 110 (16%) |
| Child development | 544 (81%) |

*Note.* *N* = 670. This item was shown only to women in the postpartum period.

**Table S2g**

*Feature Preferences for a hypothetical mobile application (Components derived from PCA)*

|  | Mean | *SD* |
| --- | --- | --- |
|  | Mean | *SD* |
| Component 1: Data management & exchange with professionals |  |  |
| All health data in one app | 3.84 | 1.11 |
| Appointment scheduling with professionals | 3.75 | 1.18 |
| Video consultation with a professional | 3.09 | 1.27 |
| Prescriptions for medications/orders in the app | 3.81 | 1.21 |
| Tracking my health data (e.g. blood pressure) | 3.41 | 1.20 |
| Automatic sharing of data with professionals | 3.59 | 1.19 |
| Component 2: Healthy behaviours |  |  |
| Reminders for health-promoting behaviours (e.g. eating, exercise) | 2.96 | 1.24 |

*Note.* *N* = 1,160.

**Table S2h**

*Trust in providers of digital health technologies*

|  | *n* (%) |
| --- | --- |
| Private provider | 44 (5.9%) |
| Health insurance company | 74 (10.0%) |
| Federal/State government | 214 (29%) |
| University/College | 86 (12%) |
| Professional association | 54 (7.3%) |
| Hospital | 269 (36%) |

*Note.* *N* = 740. This item was shown only to women who mentioned data protection concerns or the potential drawbacks of automated computer-based decisions as significant disadvantages of healthcare digitalization (cf. Table S2i).

**Table S2i**

*Perceived advantages and disadvantages of increasing use of digital technology in healthcare (Components derived from PCA)*

|  | *n* (%) |
| --- | --- |
| Component 1: Advantages |  |
| Simplified information exchange | 843 (73%) |
| No unnecessary travel to healthcare professionals | 529 (46%) |
| Reduced risk of on-site infection (e.g. COVID-19) | 194 (17%) |
| Faster access to and overview of personal health data | 851 (73%) |
| Monitoring own health status at home | 313 (27%) |
| Component 2: General disadvantages |  |
| Poor internet connection or technical issues | 405 (35%) |
| Impersonal contact with healthcare professionals | 824 (71%) |
| Difficulty in reaching healthcare professionals | 348 (30%) |
| Having to collect data oneself | 258 (22%) |
| Being at the mercy of computer decisions | 435 (38%) |
| Component 3: Disadvantages related to data protection |  |
| Insufficient protection of personal health data | 639 (55%) |

*Note.* *N* = 1,160.
